# RNA sequence analysis of differentially expressed genes in left atrial appendage thrombus

**DOI:** 10.1007/s11239-025-03184-1

**Published:** 2025-10-05

**Authors:** Junji Maeda, Motoki Furutani, Shunsuke Miyauchi, Mika Nakashima, Naoki Ishibashi, Takumi Sakai, Naoto Oguri, Shogo Miyamoto, Sho Okamura, Yousaku Okubo, Takehito Tokuyama, Noboru Oda, Taiichi Takasaki, Shinya Takahashi, Hidenori Aizawa, Daichi Shigemizu, Yukiko Nakano

**Affiliations:** 1 Department of Cardiovascular Medicine, Graduate School of Biomedical and Health Sciences, Hiroshima University, 1-2-3 Kasumi, Minami-ku, Hiroshima, 734-8551 Japan; 2 Department of Surgery, Graduate School of Biomedical and Health Sciences, Hiroshima University, Hiroshima, Japan; 3 Department of Neurobiology, Graduate School of Biomedical and Health Sciences, Hiroshima University, Hiroshima, Japan; 4 Medical Genome Center, National Center for Geriatrics and Gerontology, Research Institute, Aichi, Japan

**Keywords:** Atrial fibrillation, Left atrial appendage thrombus, RNA sequencing

## Abstract

Cardioembolic stroke is a major complication of atrial fibrillation (AF). We investigated differentially expressed genes (DEGs) in the left atrial appendage (LAA) with and without LAA thrombus (LAAT) using RNA sequencing (RNA-seq). LAA tissue samples were obtained during cardiac surgery. We analyzed samples with LAAT (n = 6) and without LAAT (n = 5). Differential gene expression analysis was conducted to identify significantly altered genes. RNA-seq identified 27 differentially expressed genes (false discovery rate < 0.05,|log_2_(fold change)| >2). Among these, four DEGs—*DIRAS3*, *CYP26B1*, *PRG4*, and *ITLN1*—exhibited particularly large fold changes. Protein-protein interaction network analysis revealed two hub genes, *FKBP5* and *TUBA3D, *based on degree (≥ 30) and betweenness centrality (≥ 3000). Quantitative PCR confirmed consistent expression patterns for these genes. Furthermore, consistent results were obtained in another independent set (10 cases with LAAT and 10 cases without LAAT). Linear regression analysis, adjusted for age and gender, showed that *DIRAS3* expression was significantly associated with both the fibrosis ratio (β = 2.99, 95% confidence interval [CI] 0.22–5.75, *p* = 0.034) and NT-proBNP levels (β = 373, 95% CI 238–507, *p*= 5.71E-08). Additionally, *CYP26B1* and *TUBA3D* expression levels were significantly associated with NT-proBNP (β = 349, 95% CI 23.8–674, *p*= 0.036; β = -140, 95% CI -272 to -8.81, *p *= 0.038, respectively). We identified candidate genes potentially involved in LAAT in AF patients through RNA-seq analysis. These findings may elucidate the molecular mechanisms underlying LAAT pathogenesis.

**Graphical abstract:**

Transcriptomic analysis of LAAT in patients with AF suggested that six genes—*DIRAS3*, *CYP26B1*, *PRG4*, *ITLN1*, *FKBP5*, and *TUBA3D*—might be associated with thrombus formation. Among them, *DIRAS3* expression was positively associated with both fibrosis ratio and NT-proBNP levels. *CYP26B1* expression was also positively associated with NT-proBNP, whereas *TUBA3D* expression showed a negative association. This transcriptomic approach provides valuable insights into the pathogenesis of LAAT and highlights potential biomarkers for future investigation.

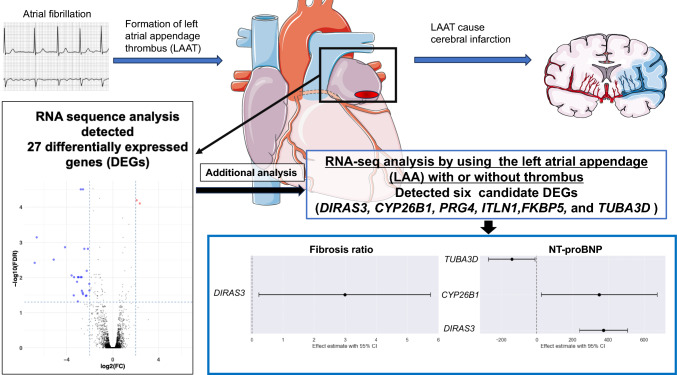

**Supplementary Information:**

The online version contains supplementary material available at 10.1007/s11239-025-03184-1.

## Highlights

- We investigated candidate genes associated with left atrial appendage thrombus (LAAT) in patients with atrial fibrillation using RNA sequencing of left atrial appendage tissue obtained during cardiac surgery.
- RNA sequencing analysis identified six key genes potentially associated with LAAT: *DIRAS3*, *CYP26B1*, *PRG4*, *ITLN1*, *FKBP5*, and *TUBA3D*.
- Among them, *DIRAS3*expression showed a positive correlation with both the degree of fibrosis and NT-proBNP levels. *CYP26B1* expression was positively correlated with NT-proBNP, whereas *TUBA3D *expression was negatively correlated.
- This transcriptomic approach provides valuable insight into the pathogenesis of LAAT formation and the identification of potential biomarkers. Further research is needed to validate these findings and assess their clinical implications.

**Supplementary Information:**

The online version contains supplementary material available at 10.1007/s11239-025-03184-1.

## Introduction

Cardiogenic embolus is mostly derived from the left atrial appendage (LAA) [1], which influences patient prognosis, quality of life, and social cost [2]. Recently, technical advances in LAA management, such as percutaneous LAA closure [3] and thoracoscopic left atrial appendectomy [4], have led to the development of effective therapeutic strategies. In this era of less invasive LAA management, it is essential to identify patients at high risk for forming LAA thrombus (LAAT). Previous studies have shown that decreased LAA function [5] and non-chicken wing LAA morphology [6] are associated with the formation of LAAT. However, the exact mechanisms of LAAT formation have not been well elucidated.

Analysis of whole messenger RNA (mRNA) expression play a crucial role in uncovering the molecular mechanisms underlying diseases. One of the most powerful tools for detecting the biomarkers or molecular functions in human tissues is whole RNA sequencing (RNA-seq). A comprehensive transcriptome analysis using next-generation sequencing has been widely applied, including paired RNA-seq analysis of left and right appendages in human [7].

In this study, RNA-seq transcriptome analyses of LAA specimens were performed to detect key genes related to the LAAT and to uncover the underlying mechanisms of LAAT.

## Methods

### Patient recruitment, LAA samples, and baseline data acquisition

This study included 31 patients with atrial AF who underwent LAA excision at the Department of Cardiac Surgery, Hiroshima University. All patients were diagnosed with AF, documented using more than one modality (e.g., 12-lead electrocardiographic testing, 24-h Holter recording, long-term Holter recording, or mobile electrocardiographic monitoring), and subjected to LAA management during cardiac surgery. Participants were classified into two groups: the LAAT and non-LAAT groups. LAAT cases were defined as patients with AF who required surgical excision of the LAA as part of treatment for LAAT. Patients with concomitant structural or valvular heart disease, other than AF and thrombus, requiring surgical intervention were excluded from the LAAT group. The non-LAAT group included patients who underwent LAA excision during surgery for valvular heart disease or coronary artery disease but did not have LAAT. Patients with end-stage renal disease requiring dialysis were excluded from both groups.

The LAA samples were obtained during cardiac surgery from 16 patients with evidence of LAAT and 15 with no clinical evidence of LAAT. Of the total samples, 11 underwent RNA sequencing, while the others were analyzed by qPCR.

Data on the patients’ baseline clinical characteristics, including their AF status, coexisting disease, and the results of the routine peripheral blood tests and echocardiography examinations, was obtained from their medical records. The CHADS_2_ score was calculated following the standard manner [8]. Moreover, paroxysmal AF was defined as recurrent AF that terminated spontaneously within seven days, whereas non-paroxysmal AF was defined as AF that persisted beyond seven days and included long-standing and permanent AF.

The presence of an LAAT was confirmed via transesophageal echocardiography (TEE) that was conducted by experienced echocardiographers before surgery using an EPIQ 7 ultrasound imaging system (Philips) and X8-2t 3-dimensional TEE transducer (Philips). An LAAT was defined as a mass with a high echogenic density, distinct from the LAA wall density, and attached to the LAA wall as well as a mass that moved independently of the LAA wall.

This study was conducted in accordance with the Declaration of Helsinki, and the study approval was obtained from the Hiroshima University Ethics Committee (approval number E-1931). Written informed consent was obtained from all patients.

### LAA excision

For patients who required LAA management alone, LAA excision was performed through thoracoscopic stand-alone left atrial appendectomy as previously reported [4]. For the other patients, LAAs were excised concomitant with other cardiac procedures. Supplemental Table 1 shows the primary cardiac disease and procedures. After resection, the LAA is thoroughly washed with saline, so thrombi and blood clots are not included in the analysis samples. The two 10 × 10 mm sections from the distal side of each LAA specimen were randomly resected and fixed in formalin and paraffin-embedded. The remaining specimens were snap-frozen in liquid nitrogen immediately and stored at a temperature of − 80 °C.

### Quantification of LAA fibrosis

The quantification of the degree of LAA fibrosis was performed as previously reported [9]. In brief, the two formalin-fixed and paraffin-embedded LAA Sects. (4.5-μm thick) were deparaffinized and subjected to Azan–Mallory staining. The two microscopic fields with 200 × magnification were randomly selected and imaged for each LAA section. After the manual removal of the perivascular tissue, epicardium, endocardium, and fatty tissue, the red (myocardium) and blue areas (fibrosis) in each image were measured using the BZ-X800 Analyzer software (Keyence, Osaka, Japan). Moreover, the degree of the LAA fibrosis (%) in each microscopic field was measured by dividing the fibrosis area by the fibrosis area plus the myocardium area and multiplying by 100. Furthermore, the average of the four microscopic fields was calculated. These procedures were performed by experienced pathologists who had no access to the clinical data.

### RNA extraction

From the frozen LAA specimens, 25 mg sections were randomly resected from the distal side of the LAA. Each section was homogenized with zirconia-silica beads using a bead beater homogenizer (µT-12, TAITEC, Saitama, Japan). After homogenization, the RNA was extracted using the easy-spin Total RNA Extraction Kit (iNtRON Biotechnology, Seongnam, Korea) in accordance with the manufacturer’s instructions.

### RNA-seq data analysis

RNA-seq–based transcriptome profiling was performed by the Beijing Genomics Institute (Wuhan) using the BGISEQ platform [10]. Mapping to a human reference genome (GRCh37) with STAR (ver. 2.5.2b) was performed using clean sequenced reads from BGISEQ. The read counts for each gene were used to measure the RSEM program (version 1.3.0 [https://deweylab.github.io/RSEM]). Differential gene expression analysis was performed using the edgeR package (ver. 3.8.1) program (https://bioconductor.org/packages/release/bioc/html/edgeR.html) after the read counts from each sample were combined into a count file. Moreover, we filtered by minimum read counts using the “filterByExpr” function of the edgeR package. The “caclNormFactors” function in the edgeR package was used to obtain the TMM (trimmed mean of M-values) normalization factors to account for library sizes. We applied the “exactTest” function in the edgeR package to obtain differentially expressed genes (DEGs) between the samples from patients with and without LAAT. The DEGs were defined as genes with a false discovery rate (FDR) of < 0.05, |log_2_(fold change [FC])|> 2 and a normalized transcript per million (nTPM) of ≥ 1 in the heart muscle from the human protein atlas database (https://www.proteinatlas.org). The FDR values were measured using the Benjamini–Hochberg method. The TPM was obtained using RSEM (version 1.3.0 [https://deweylab.github.io/RSEM]) after utilizing STAR to align the RNA-seq reads to the human reference genome.

### Gene ontology molecular function term enrichment analysis

A Gene Ontology (GO) term enrichment analysis was performed using the significant DEGs to assess molecular functions. Statistically significant GO terms (FDR < 0.05) were investigated based on the DEGs using the Database that includes Annotation, Visualization, and Integrated Discovery (DAVID, version 2023q4; https://david.ncifcrf.gov/).

### Protein–protein interaction network analysis

To identify key genes and critical gene modules, a protein–protein interaction (PPI) network analysis was conducted using NetworkAnalyst (https://www.networkanalyst.ca), integrating data from the DifferentialNet database (https://netbio.bgu.ac.il/diffnet/). Tissue-specific interaction data corresponding to the heart atrial appendage were employed, with a filter level of 15 applied. The PPI network was visualized using Cytoscape (version 3.10.3, http://www.cytoscape.org/).

### Validation of RNA-seq results by quantitative pcr and in an independent sample set

Using the ReverTra Ace qPCR RT kit (TOYOBO, Osaka, Japan), cDNA was synthesized. The following conditions were used for reverse transcription: the first step to prepare the mixture was one cycle of 65 °C for 5 min and cooling immediately, and the second step was one cycle of 37 °C for 15 min, one cycle of 50 °C for 5 min, and one cycle of 98 °C for 5 min. A real-time PCR analysis was performed using QuantStudio5™ Real-Time PCR Systems 384-well plates (Thermo Fisher Scientific, Waltham, MA, USA) and PowerUp™ SYBR™ Green Master Mix (Thermo Fisher Scientific, Waltham, MA, USA). Supplemental Table 2 shows the target genes and their corresponding primers. The real-time PCR conditions were as follows: one cycle of 50 °C for 2 min, one cycle of 95 °C for 2 min, 40 cycles of 95 °C for 15 s, one cycle of 60 °C for 60 s, one cycle of 95 °C for 15 s, one cycle of 60 °C for 60 s, and one cycle of 95 °C for 15 s. Each gene was assayed in duplicate. *GAPDH* (Supplemental Table 2) was selected as a reference gene for the normalization of the target gene expression levels. Moreover, the target gene expression levels were estimated from the samples of the RNA-seq analysis. The relative gene expression levels were calculated using the 2^−ΔΔCt^ method. Additionally, we analyzed independent LAA samples, comprising of 10 samples with LAAT and 10 samples without LAAT and verified the expression levels of the candidate DEGs using the same method.

### Association between gene expression and clinical information

The correlation between the candidate DEGs and CHADS_2_ scores (congestive heart failure, hypertension, Age, diabetes, stroke) was assessed by Spearman's rank correlation coefficient. Additionally, the associations between the expression of candidate DEGs and clinical information (fibrosis ratio and NT-proBNP) were evaluated using linear regression analysis.

### ROC curve–based assessment of diagnostic performance

The AUC for LAAT using all six of the candidate genes, we performed receiver operating characteristic (ROC) curve analysis and calculated the area under the curve (AUC). Relative gene expression levels, derived using the 2^−ΔΔCt^ method, were used as parameters for both RNA-seq and validation samples. LAAT cases were primarily composed of patients with persistent AF (all LAAT cases in both the RNA-seq and validation cohorts had persistent AF), whereas non-LAAT cases had fewer cases of persistent AF (one-fifth of the RNA-seq cohort and 5 out of 10 cases in the validation cohort had persistent AF) and more cases of paroxysmal AF (Supplementary Table 1 and Supplementary Table 3). Therefore, ROC curve analyses of AUC were conducted not only for LAAT status but also for AF type (persistent vs. paroxysmal) to evaluate differences in candidate gene expression between the two AF phenotypes.

### Expression patterns of candidate DEGs based on the Heart Cell Atlas

To investigate the cell type–specific expression patterns of the DEGs identified by our RNA-seq analysis, we utilized publicly available single-cell RNA-seq data from the Heart Cell Atlas (https://www.heartcellatlas.org/) [11] to estimate the expression and localization of the candidate DEGs across various cardiac cell populations.

### Statistical analysis

Continuous variables are presented as mean ± Standard deviation (SD), and categorical variables are presented as counts (percentages). Statistical analysis was performed using Python version 3.8.12 and R version 4.3.1. For clinical data, continuous variables were compared using the Mann–Whitney U test when the data did not follow a normal distribution, and Welch’s t-test when the data followed a normal distribution. Categorical variables were compared using Fisher’s exact test. The correlation between the CHADS_2_ scores and gene expression was estimated using Spearman's rank correlation coefficient. Association between clinical information and gene expression were assessed using linear regression adjusted for age and gender. A *P* value of < 0.05 was considered statistically significant for clinical items, and an FDR of < 0.05 was considered significant in the RNA-seq analysis. The ROC curve and AUC were estimated from logistic regression models using the R package “pROC”.

## Results

### Clinical information

Six patients with evidence of LAAT (33.33% female, mean age 69.97 ± 9.07 years) and five patients without clinical evidence of LAAT (40% female, mean age 70.60 ± 11.46) were enrolled in this study. Table 1 and Supplemental Table 1 show the baseline demographic data of the participants. Persistent AF was significantly more common in the LAAT participants, while NT-proBNP was significantly higher in participants with LAAT compared to those without (*P* < 0.05). No statistical differences were observed between other clinical factors and the LAA fibrosis ratio (Table 1).

**Table 1:** Demographic data of study participants

Items	LAAT (n = 6)	without LAAT (n = 5)	P value
Gender (Female)	2	2	1*
Persistent atrial fibrillation	6	1	0.015*
Past history of stroke (No = 0, Yes = 1)	2	1	1*
Medication of anticoagulant (No = 0, Yes = 1)	6	4	0.45*
Age	69.67 ± 9.07	70.6 ± 11.46	0.54^†^
Body mass index	23.68 ± 2.81	21.7 ± 1.21	0.33^†^
CHADS2 score	3.33 ± 1.37	1.80 ± 1.10	0.11^†^
White blood cell	7641.67 ± 3161.86	6490.00 ± 3290.81	0.54^†^
Neutrophil	5600.00 ± 3135.03	3620.00 ± 1478.21	0.43^†^
Lymphocyte	1575.00 ± 490.42	2382.00 ± 1724.3	0.93^†^
C-reactive protein	0.18 ± 0.2	0.15 ± 0.13	1^†^
Hemglobin	14.7 ± 1.38	12.2 ± 2.23	0.08^†^
Platelet	216.67 ± 48.72	238.2 ± 65.37	0.41^†^
Hemoglobin A1c	6.18 ±.58	6.22 ± 1.68	0.85^†^
Triglycerides	199.33 ± 73.36	129.8 ± 79.08	0.18^†^
Low density Lipoprotein cholestrol	99.5 ± 19.72	90.4 ± 11.15	0.27^†^
High density Lipoprotein cholestrol	53. ± 16.85	57.00 ± 23.44	0.93^†^
Blood urea nitorgen	18.58 ± 5.74	15.78 ± 4.57	0.46^†^
Creatinine	0.96 ± 0.29	0.83 ± 0.29	0.66^†^
eGFR	59.33 ± 18.15	66.6 ± 12.6	0.71^†^
NT-proBNP	1288.5 ± 667.79	346.00 ± 223.59	**4.33E-03**^**†**^
TTE EF	45.00 ± 17.41	54.00 ± 14.28	0.58^†^
LAA fibrosis ratio	14.94 ± 10.40	9.28 ± 3.01	0.66^†^

LAAT: left atrial appendage thrombus, LAA: left atrial appendage, eGFR: estimated glomerular filtration rate, TEE: transthoracic echocardiogram, EF: ejection fraction.

^*^Fisher's exact test

^†^Mann–whitney U test

### Detection of DEGs

**Fig. 1:**
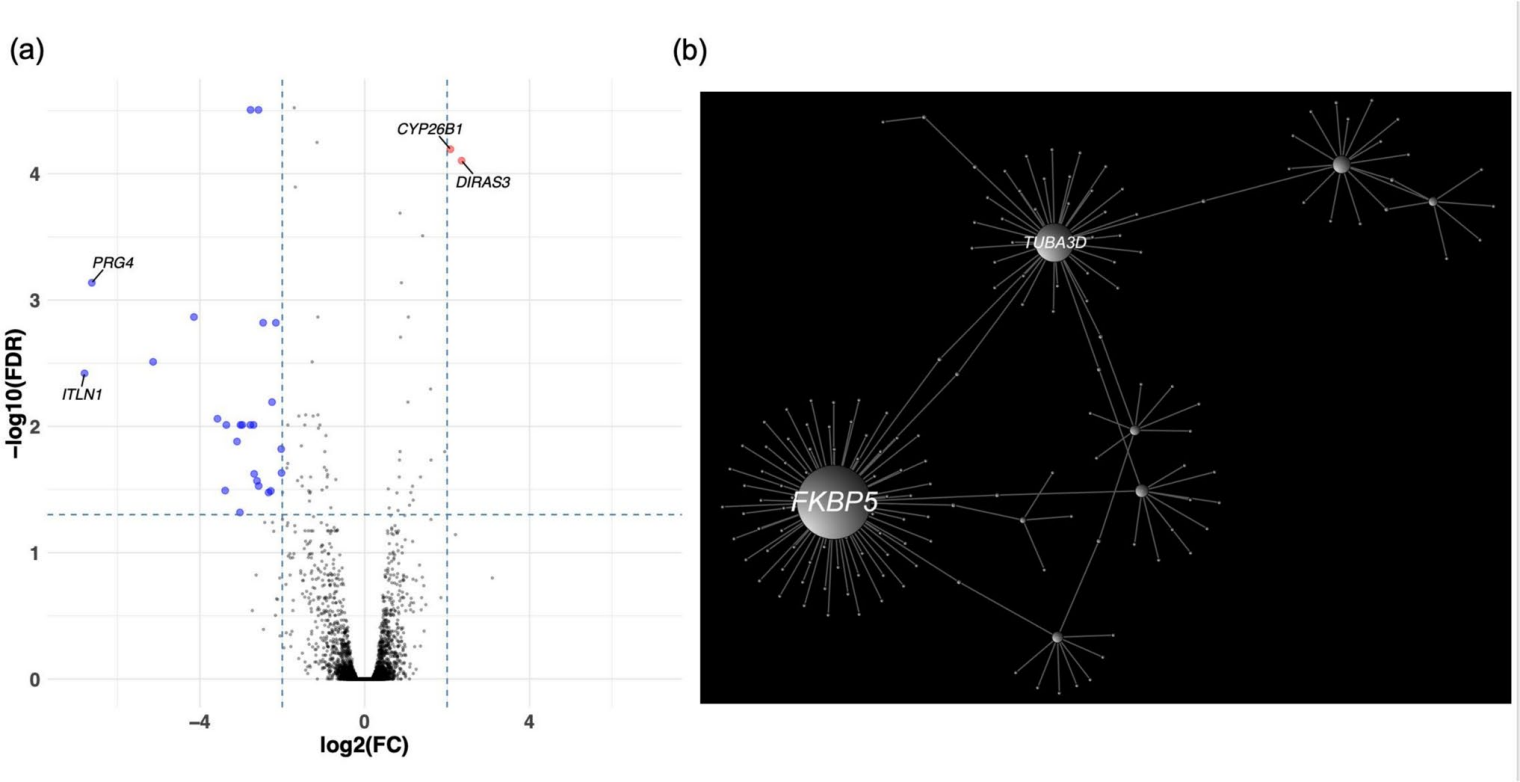
Results of RNA-seq analysis. (**a**) DEG detection by RNA-seq. Each point represents a DEG. The blue and red dots represent the downregulated and upregulated DEGs, respectively. (**b**) PPI network analysis. The hub genes were defined as genes with a DC of ≥ 30 and BC of ≥ 3000. DEG, differentially expressed gene; PPI, protein–protein interaction; BC, betweenness of centrality; DC, degree of centrality; FC, fold change; FDR, false discovery ratio

RNA-seq analysis was performed on all LAA samples using an average of > 48.7 million high-quality read sequences, with > 96.21% uniquely mapped to the human reference genome (GRCh37) (Supplemental Table 4). A total of 27 significant DEGs were identified from the 13,722 analyzed genes, based on the criteria of an FDR < 0.05, |FC|> 2, and a nTPM ≥ 1 in heart muscle, according to the Human Protein Atlas. Among these, 2 genes were upregulated and 25 were downregulated in the LAAT samples. Notably, four DEGs—*DIRAS3*, *CYP26B1*, *PRG4*, and *ITLN1*—exhibited particularly large fold changes and were selected for further analysis (Fig. 1a, Table 2, and Supplemental Table 5).

**Table 2:** Most significant DEGs

Ensembl Gene iD	Gene Symbol	logFC	logCPM	nTPM in heart tissue	P value	FDR
ENSG00000003137	*CYP26B1*	2.08	2.96	6	2.6E-08	6.41E-05
ENSG00000162595	*DIRAS3*	2.35	1.82	11.4	3.9E-08	7.89E-05
ENSG00000116690	*PRG4*	−6.62	7.75	18.7	6.1E-07	7.30E-04
ENSG00000179914	*ITLN1*	−6.8	7.67	125	6.2E-06	0.0038

FC: fold change, CPM: count per milion, nTPM: normalized transcripts per million, FDR: false discovery rate

### GO term enrichment analysis and PPI network analysis

There was no GO molecular function term with an FDR < 0.05. Subsequently, we performed a PPI network analysis using the 27 DEGs, utilizing tissue-specific interaction data for heart atrial appendage from DifferentialNet database (https://netbio.bgu.ac.il/labwebsite/software/differentialnet/). The PPI network was comprised of 165 nodes and 169 edges. We identified the most highly ranked hub genes in terms of network topology measures of the degree of centrality (DC) and betweenness centrality (BC). Two hub genes were detected with DC ≥ 30 and BC ≥ 3000: *FKBP5* (DC = 75, BC = 9989.08), and *TUBA3D* (DC = 38, BC = 7993.42) (Fig. 1b and Table 3).

**Table 3:** Hub genes

Ensembl Gene ID	Gene Symbol	logFC	logCPM	Regulation	nTPM in heart muscle	Degree	Betweenness	Pvalue	FDR
ENSG00000096060	*FKBP5*	−2.16	5.52	DOWN	90.5	75	9989.08	0.00000195	0.0015
ENSG00000075886	*TUBA3D*	−3.57	1.51	DOWN	47.7	38	7993.42	1.99E-05	0.0087

FC: fold change, CPM: count per milion, nTPM: normalized transcripts per million, FDR: false discovery rate

### Verification of the quantitative PCR assay

**Fig. 2:**
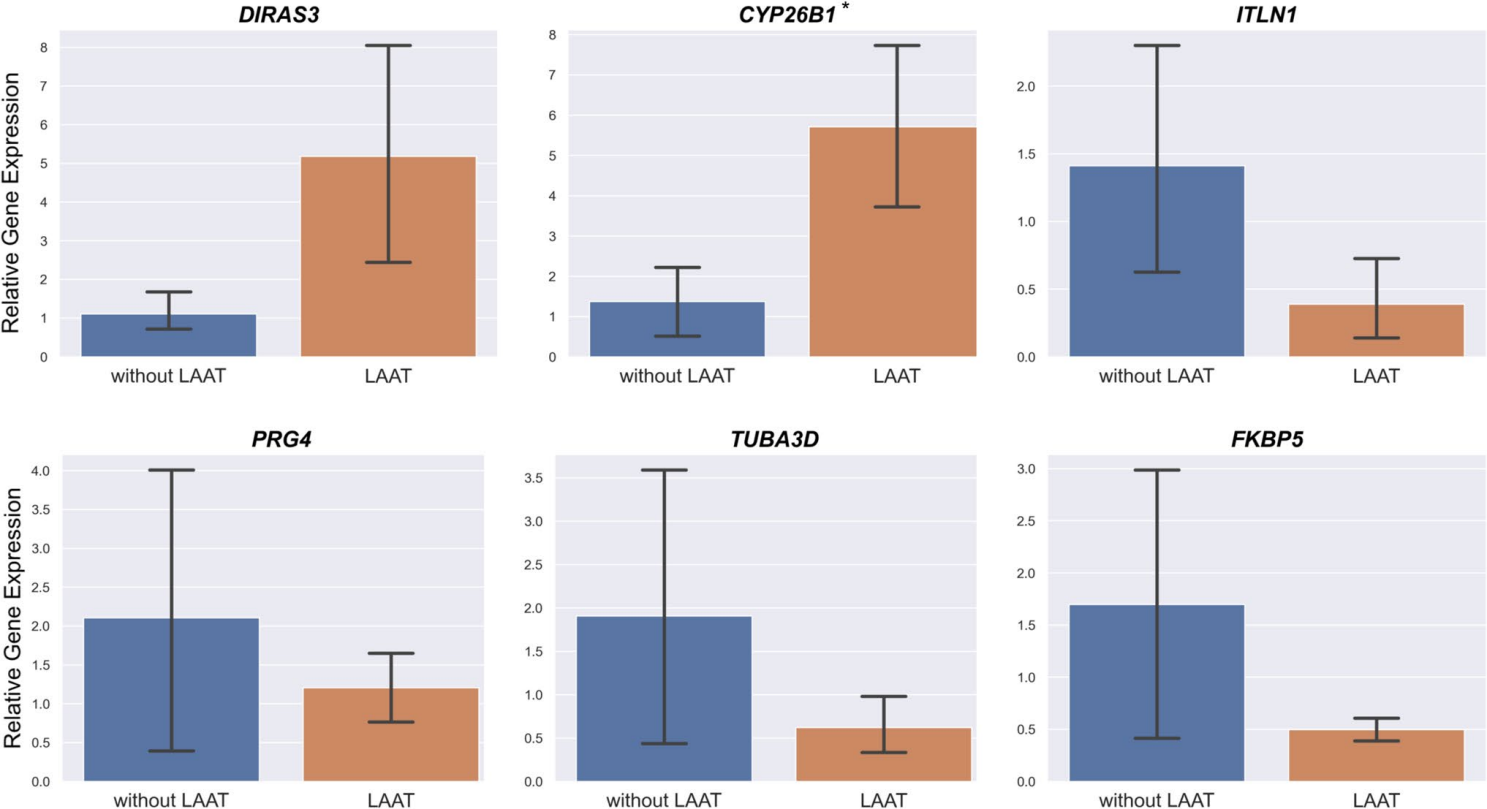
Quantitative PCR verification of the RNA-seq data The error bars in the quantitative PCR indicate standard errors.The mean relative gene expression ratios (LAAT/without LAAT) were as follows: 4.65 for *DIRAS3,* 4.15 for *CYP26B1*, 0.28 for *ITLN1*, 0.57 for *PRG4*, 0.33 for *TUBA3D*, and 0.29 for *FKBP5*. *: *P* < 0.05 LAAT, left atrial appendage thrombus

Quantitative PCR analysis was performed to validate six genes identified by our RNA-seq analysis: *DIRAS3, CYP26B1, ILTN1, PRG4, FKBP*, and *TUBA3D*. The mean relative gene expression ratios (LAAT/without LAAT) were as follows: 4.65 for *DIRAS3,* 4.15 for *CYP26B1*, 0.28 for *ITLN1*, 0.57 for *PRG4*, 0.33 for *TUBA3D*, and 0.29 for *FKBP5*. These gene expressions were consistent with the RNA-seq results, supporting the reliability of the transcriptomic findings (Fig. 2). The expression levels of the candidate DEGs were evaluated in the independent LAA sample sets, consisting of 10 cases with LAAT and 10 cases without LAAT (Supplementary Table 3). The mean relative gene expression ratios (LAAT/without LAAT) of the independent LAA samples were as follows: 1.92 for *DIRAS3,* 1.86 for *CYP26B1*, 0.064 for *ITLN1*, 0.042 for *PRG4*, 0.60 for *TUBA3D*, and 0.035 for *FKBP5*. The qPCR results using these samples also showed results consistent with the expression patterns observed in the RNA-seq analysis Supplementary Fig. 1.

### Association study between clinical information and gene expression

No significant DEGs were correlated with CHADS2 scores. However, *DIRAS3* expression showed a significant positive correlation with Heart failure (spearman’s *r* = 0.66, *P* = 0.030) (Supplementary Table 6). Linear regression analysis further revealed that *DIRAS3* expression was positively associated with the fibrosis ratio (β = 2.99, 95% confidence interval [CI] 0.22–5.75, *p* = 0.034) and NT-proBNP (β = 373, 95% CI 238–507, *p* = 5.71E-08). Additionally, *CYP26B1* and *TUBA3D* expression levels were significantly associated with NT-proBNP (β = 349, 95% CI 23.8–674, p = 0.036; β = −140, 95% CI −272 to −8.81, *p* = 0.038, respectively) (Supplementary Table 7).

### ROC curve–based assessment of diagnostic performance

The AUC for LAAT using all six candidate genes was 0.98 (95% CI, 0.93–1.00). The AUC values for each individual gene were as follows: *DIRAS3*, 0.81; *CYP26B1*, 0.80; *PRG4*, 0.72; *ITLN1*, 0.79; *FKBP5*, 0.72; and *TUBA3D*, 0.63 (Supplementary Fig. 2). The AUC for AF type (persistent or paroxysmal) using all six candidate genes was 0.95 (95% CI, 0.88–1.00). Among those, the AUC values for each individual gene were as follows: *DIRAS3*, 0.84; CYP26B1, 0.88; *PRG4*, 0.61; *ITLN1*, 0.66; *FKBP5*, 0.69; and *TUBA3D*, 0.62 (Supplementary Fig. 3).

Although both AUCs were high, the genes *DIRAS3*, and *CYP26B1* which are associated with fibrosis or NT-proBNP (Supplementary Table 7), appeared to be somewhat effective for distinguishing AF types.

### Expression patterns of candidate DEGs based on the Heart Cell Atlas 

We used the publicly available Heart Cell Atlas (https://www.heartcellatlas.org/) to investigate the cell type–specific expression patterns of the candidate DEGs (*CYP26B1*, *DIRAS3*, and *TUBA3D*) identified in our RNA-seq analysis [11]. *CYP26B1* was predominantly expressed in fibroblasts, mesothelial cells, and adipocytes. *DIRAS3* is mainly expressed in both atrial and ventricular cardiomyocytes, as well as in lymphatic endothelial and mesothelial cells. *TUBA3D* expression was observed in ventricular cardiomyocytes (Supplementary Fig. 4).

## Discussion

AF is a major risk factor for stroke; intracardiac thrombi are more likely to form in patients with AF, and more than 90% of these thrombi form within the LAA. Virchow’s triad (hypercoagulability, hemodynamic changes, and endothelial injury) has been implicated in thrombus formation [12]. Recently, atrial cardiomyopathy, which is caused by atrial structural and electrophysiological remodeling has been considered an important risk factor of cardioembolic stroke [13]. Previously, we suggested that LAA fibrosis and endocardial endothelial damage are associated with LAAT and stroke using a histological approach and confirmed the relationship between LAAT and stroke within the atrial cardiomyopathy context [14]. The present study aimed to focus on not only the histological approach but also the approach for detecting the molecular mechanism via RNA-seq analysis to provide a better understanding of LAAT.

We identified 27 DEGs and focused on four DEGs with large fold changes (i.e., *DIRAS3*, *CYP26BI*, *PRG4*, and *ITLN1*). In addition, we detected two hub genes from PPI analysis (*FKBP5* and *TUBA3D*). The expression of these six genes could be reproduced by qPCR. Of them, *DIRAS3* was significantly associated with a past history of heart failure and linear regression analysis revealed that the expression of *DIRAS3* was significantly associated with fibrosis ratio and NT-pro BNP. The expression of *CYP26B1* and *TUBA3D* were also associated with NT-proBNP.

These DEGs may contribute to thrombus formation and stabilization through mechanisms shared with cancer biology and immune regulation, such as autophagy [15–18], inflammation [19–22], oxidative stress [23, 24], ion channel regulation [25–27], and cytoskeletal remodeling [28, 29]. Indeed, stress responses in myocardial tissue resemble signaling mechanisms long established in cancer biology and immune regulation. For example, in cancer, inhibition of the mTOR pathway under nutrient deprivation supports tumor cell survival through autophagy [15], while in the immune system, the same mTOR–autophagy signaling regulates immune cell differentiation [16]. In the heart, inhibition of the mTOR pathway under hemodynamic or metabolic stress induces autophagy, which promotes myocardial fibrosis and remodeling [17, 18]. Furthermore, activation of the NLRP3 inflammasome and subsequent IL-1β signaling contribute to tumor progression within the cancer microenvironment [19] and modulate innate immune responses [20]. In the heart the same pathway promotes atrial fibrosis and heart failure [21, 22]. Oxidative stress mediated by mitochondrial ROS is a common mediator activating MAPK and NF-κB pathways in cancer and immune cells [23], while in the heart, it induces fibrotic remodeling leading to heart failure [24]. Ion channels are crucial in cancer and immunity, where dysregulated calcium, potassium, and chloride channels promote cell proliferation, migration, and invasion [25, 26]. They are also important in cardiovascular disease, including fibrosis [27]. Furthermore, cytoskeletal remodeling enables tumor invasion [28], but impairs contractile function under cardiac stress [29]. These parallels underscore that the molecular pathways involved in cardiac stress are not unique, but rather converge with conserved mechanisms widely characterized in oncology and immunology.

*DIRAS3* is considered to be associated with the regulation of autophagy. *DIRAS3* (DIRAS family GTPase 3) is tumor suppressor gene which inhibit RAS function [30]. Not only tumor, Asim Ejaz et al. reported that DIRAS3 in human white adipose progenitor cells inhibits adipogenesis and activated autophagy through Akt-mTOR inhibition, then incapacitate cellular senescence and convince extension of lifespan [31]. With regard to the heart, Chuanjun Zhuo et al. reported that high glucose increased DIRAS3 expression in cardiomyocytes, and DIRAS3 induced autophagy by inhibiting mTOR signaling, which cause diabetic cardiomyopathy [32]. Autophagy plays an important role for cardiac fibrosis [33–35]. S Ghavami et al. reported that autophagy is a regulator of fibrogenesis in human atrial myofibroblasts [36]. In this study, the upregulation of *DIRAS3* in the LAA with thrombus might indicate the enhancement of autophagy in the LAA, and the positive correlation between ratio of LAA fibrosis and expression of *DIRAS3* was also detected. Previously, we revealed the histological evidence of the association between LAA fibrosis and endocardial endothelial damage associated with LAAT and ischemic stroke [14]. The upregulation of *DIRAS3* in the LAA may play an important role for thrombus formation and stabilization through fibrosis of the LAA. Autophagy also associated with cause of heart failure [33, 37] and cardiac aging which causes hypertrophy, dysfunction of mitochondria as well as fibrosis [38]. *DIRAS3* was also correlated with NTproBNP. The upregulation of *DIRAS3* in the LAA with thrombus might contribute to heart failure such as atrial myopathy through autophagy in the LAA and fibrosis.

The expression of *DIRAS3* was observed in the atrial and ventricular cardiomyocytes, mesothelial cell and fibroblast from the Heart Cell Atlas data (https://www.heartcellatlas.org/) [11]. These results implicated that expression of *DIRAS3* in the LAA could be the potential key to assessing the risk of stroke in AF patients.

*CYP26B1* is considered to be associated with inflammation and oxidative stress. Previous study reported that *CYP26B1* (cytochrome P450, family 26 subfamily B, polypeptide 1) related to atherosclerosis through retinoic acid catabolism [39]. Activation of retinoic acid receptors upregulates the antiatherogenic genes in macrophages [40], and reduces inflammation [41], and vascular cell proliferation, and coagulation [42, 43]. Oleysta Kruvispitskata et al. revealed that atherosclerotic arteries had higher levels of CYP26B1 [39], and they also revealed that the strongest expression of CYP26B1 in macrophage-rich inflammatory lesions [39]. From our investigation, the upregulation of *CYP26B1* in the LAA may also indicate the high inflammation levels of this lesion and may contribute to LAAT.

Previous studies using Helicobacter pylori (HP) provided important clues from the perspectives of oxidative stress and inflammation [44, 45]. Notably, HP infection of gastric cancer cells identified candidate infection-related genes and demonstrated that HP promotes the migratory ability of gastric cancer cells [44], suggesting a mechanism by which chronic inflammation alters cellular adhesion and motility. That finding implies that chronic inflammation in atherosclerotic arteries may similarly impair adhesion and motility through endothelial injury within Virchow’s triad. Terminalia chebula has been reported to counter HP infection by disrupting the bacterial structure, inhibiting key pathogenic proteins, and modulating inflammasome and ER-stress pathways [45]. Taken together, amelioration of chronic inflammation may represent a therapeutic strategy for LAAT. Although we were unable to find a significant relationship between the expression of *CYP26B1* and the degree of fibrosis, the Heart Cell Atlas data (https://www.heartcellatlas.org/) shows that *CYP26B1* is highly expressed in fibroblasts [11], and it is possible that *CYP26B1* is also related to fibrosis.

*TUBA3D* is considered to be associated with channel regulation, and cytoskeletal remodeling. *TUBA3D* encodes a member of the alpha-tubulin family, a key structural component of microtubules. Microtubules are dynamic cytoskeletal polymers composed of alpha- and beta-tubulin heterodimers, along with microtubule-associated proteins. They play essential roles in maintaining cellular architecture, facilitating intracellular transport, and forming the mitotic spindle during cell division [46]. A previous study, using the Gene Expression Omnibus dataset (GSE116250) obtained from human left ventricles, showed that the expression of *TUBA3D* in dilated cardiomyopathy-induced heart failure is significantly decreased compared to non-failing donors [47]. In fact, microtubule networks (MTNs) are important factors regulating the transport of Kv1.5 channels to atrial cell membranes, and Kv1.5 functions as an essential potassium channel in AF [48, 49]. Beyond ion channels, microtubules are also involved in the regulation of gap junction channels such as Cx43, which mediate the transmission of intercellular action potentials [50] and are implicated in the pathophysiology of AF [51]. A previous study using dogs subjected to atrial tachy-pacing revealed that activation of histone deacetylase 6 (HDAC6) disrupts α-tubulin proteostasis and microtubule integrity, leading to remodeling and contractile dysfunction. Inhibition of HDAC6 protected against these pathological changes, suggesting that it could be a potential therapeutic target. Downregulation of *TUBA3D* in the atrial appendage suggests some microtubule dysfunction and potential involvement in thrombus formation and stabilization via these mechanisms [52].

In the field of oncology, there are examples of repurposing drugs not originally developed for fibrosis or cardiovascular disease. For example, local anesthetics have been reported to suppress tumor growth and proliferation in cancer treatment by reducing reactive oxygen species levels and increasing autophagy extension markers [53]. Our research suggests that atrial fibrosis is a key factor in the thrombosis process. Recent studies in oncology have revealed that cancer-associated fibroblasts communicate with tumor cells via extracellular vesicles (EVs) carrying proteins and miRNAs that drive stromal remodeling and treatment resistance [54]. Similarly EV–miRNA signaling plays a crucial role in atrial disease. Exosomal miR-224-5p derived from atrial fibroblast promotes electrical remodeling by suppressing *CACNA1C* [55]; altered EV-associated miRNA profiles are observed in AF patients [56]; and EVs derived from adjacent epicardial fat can induce atrial fibrosis and myopathy [57]. These observations support a fibroblast-driven, EV-mediated atrial remodeling axis that may intersect with thrombogenesis in the LAA. In the LAA, platelet-derived EVs exhibit a pro-coagulant effect and their concentration increases in AF. Taken together, this evidence suggests that fibroblast–EV–miRNA pathways represent a unifying mechanism linking atrial fibrosis and thrombus biology, warranting translational research as circulating biomarkers and therapeutic targets. Furthermore, recent work by Liu et al. in oncology, has demonstrated that fibrosis can be approachable not only histologically but also through biomarker-guided therapeutic strategies [58]. From this perspective, the genes we identified may serve as candidate biomarkers linking atrial fibrosis and thrombo-biology. Drawing an analogy from tumor–stroma interactions, these markers could enhance the biological and clinical significance of our findings and support the development of precision-based approaches for atrial disease.

Furthermore, from a translational perspective, cancer research provides valuable precedents. For instance, local anesthetics have been studied for their ability to modulate autophagy, adhesion, and ion channel activity [59, 60]. The genes we identified are also involved in these mechanisms (*DIRAS3*: autophagy, *CYP26B1*: adhesion through chronic inflammation, *TUBA3D*: channel regulation via microtubules), suggesting that similar approaches may be effective in atrial fibrosis and thrombosis prevention. In the context of drug repurposing, metformin has emerged as a promising candidate for AF which inhibits oxidative stress, inflammation and remodeling [61]. Additional therapeutic strategies include antifibrotic agents targeting the renin–angiotensin–aldosterone system, modulators of novel pathways such as TGF-β signaling, and cardiometabolic drugs including SGLT2 inhibitors and GLP-1 receptor agonists. Moreover, innovative approaches such as microRNA modulation and lipid nanoparticle-based therapies [62] also represent promising strategies that warrant further validation, raising expectations for advances in cardiovascular medicine in parallel with those in oncology.

In our analysis, persistent AF was more frequent in the LAAT group than the non-LAAT group. Similarly, a large-scale cohort study conducted in Japan reported an independent association between persistent atrial AF and a higher stroke incidence compared to paroxysmal AF [63]. Therefore, it remains uncertain whether the candidate DEGs identified in our study are directly involved in the pathogenesis of LAAT or merely reflect biological processes associated with persistent AF. To further elucidate this issue, we performed ROC analyses comparing persistent and paroxysmal AF and found that *DIRAS3* and *CYP26B1* were particularly useful in distinguishing AF subtypes. These findings suggest that these genes may contribute to the pathophysiology underlying persistent AF, promoting fibrosis or inflammation and inducing thrombogenicity. Further investigations are warranted to clarify these mechanisms.

### Study limitations

First, the number of samples available for RNA-seq analysis was limited. We identified candidate DEGs potentially related to the pathogenesis of LAAT, but it was initially unclear whether these findings could be applied to other LAA samples. However, results from additional independent samples verifying the expression of the candidate DEGs provided evidence supporting the reproducibility of our findings and suggested their potential relevance. Nevertheless, additional validation in a larger cohort is warranted to confirm these results.

Second, we recognize that next-generation sequencing (NGS) datasets inherently contain both biological and technical biases, which have often been emphasized in cancer research [64, 65]. In the experiment, potential biases from biological factors included: 1) tissue heterogeneity, 2) sample purity, and 3) allelic imbalance technical biases included, a) PCR amplification bias, b) sequence composition bias, c) read depth and coverage, and d) alignment and mapping errors [64]. Furthermore, conventional analysis methods tend to focus on highly expressed genes, making them prone to overlooking genes that exert subtle yet biologically significant effects [65]. Our study also employed bulk RNA-seq, which may have introduced bias into the interpretation of the LAAT's molecular profile. Therefore, single-cell RNA sequencing or spatial transcriptomics could provide a more comprehensive understanding of the underlying genetic basis of LAAT. In fact, single-cell RNA-seq analysis of atrial appendage specimens from three patients with persistent atrial fibrillation suggested the importance of complement and coagulation cascades in LAAT formation. [66]

In addition to biological bias, technical variability such as differences in sequencing platforms must also be considered. To mitigate these limitations, emerging computational approaches—including the application of generative adversarial networks (GANs)—may compliment RNA-seq datasets, reduce analytical noise, and facilitate the identification of clinically relevant biomarkers [67]. Such approaches may be useful for further interpretation in genome analysis.

Despite these limitations, our study provided insights into the mechanisms underlying LAAT formation and suggests the clinical relevance of potential molecular targets. Liquid biopsy has been established as a powerful tool in oncology for detecting circulating nucleic acids and proteins as dynamic biomarkers [68], and its utility has also been demonstrated in cardiovascular disease [69]. A similar approach may hold promise for diagnosing atrial fibrosis and thrombosis. In this study, we had not yet verified whether *DIRAS3, CYP26B1, PRG4, ITLN1, FKBP5,* and *TUBA3D* could be detected in circulating fractions such as plasma-derived RNA or extracellular vesicles. If detectable, we would like to verify their potential for incorporation into a minimally invasive assay. Future studies confirming their presence in cell-free RNA or exosomal proteins would also be useful for establishing the foundation for clinical application [70].

## Conclusion

In this study, we highlighted potential candidate genes associated with LAAT in AF patients using RNA-seq analysis. These genes may contribute to the pathogenesis of LAAT and serve as potential biomarkers. In this study, all LAAT samples were derived from patients with persistent AF, whereas non-LAAT samples included few persistent AF patients. Considering this, these biomarkers may be associated not only with thrombogenesis but also with the progression of AF. Further validation may help to elucidate the potential clinical relevance of these genes and deepen our understanding of the mechanisms underlying LAAT formation.

## Supplementary Information

Below is the link to the electronic supplementary material.

Supplementary file1 (PDF 200 KB)

Supplementary file2 (PDF 373 KB)

Supplementary file3 (PDF 365 KB)

Supplementary file4 (PDF 309 KB)

Supplementary file5 (XLSX 11 KB)

Supplementary file6 (XLSX 10 KB)

Supplementary file7 (XLSX 11 KB)

Supplementary file8 (XLSX 11 KB)

Supplementary file9 (XLSX 11 KB)

Supplementary file10 (XLSX 11 KB)

Supplementary file11 (XLSX 10 KB)

## Data Availability

The data that support the findings of this study are not publicly available due to privacy and ethical restrictions but are available from the corresponding author upon reasonable request.The Graphical Abstract was designed using Servier Medical Art images (https://smart.servier.com).
