# Supplementary material for: RNA sequence analysis of differentially expressed genes in left atrial appendage thrombus
Source: J Thromb Thrombolysis. 2025 Oct 5;59(2):437–49. doi: 10.1007/s11239-025-03184-1 (PMC13018052; doi:10.1007/s11239-025-03184-1)
Supplement: Supplementary file 1 — Supplementary file1 (PDF 200 KB) [file 11239_2025_3184_MOESM1_ESM.pdf]

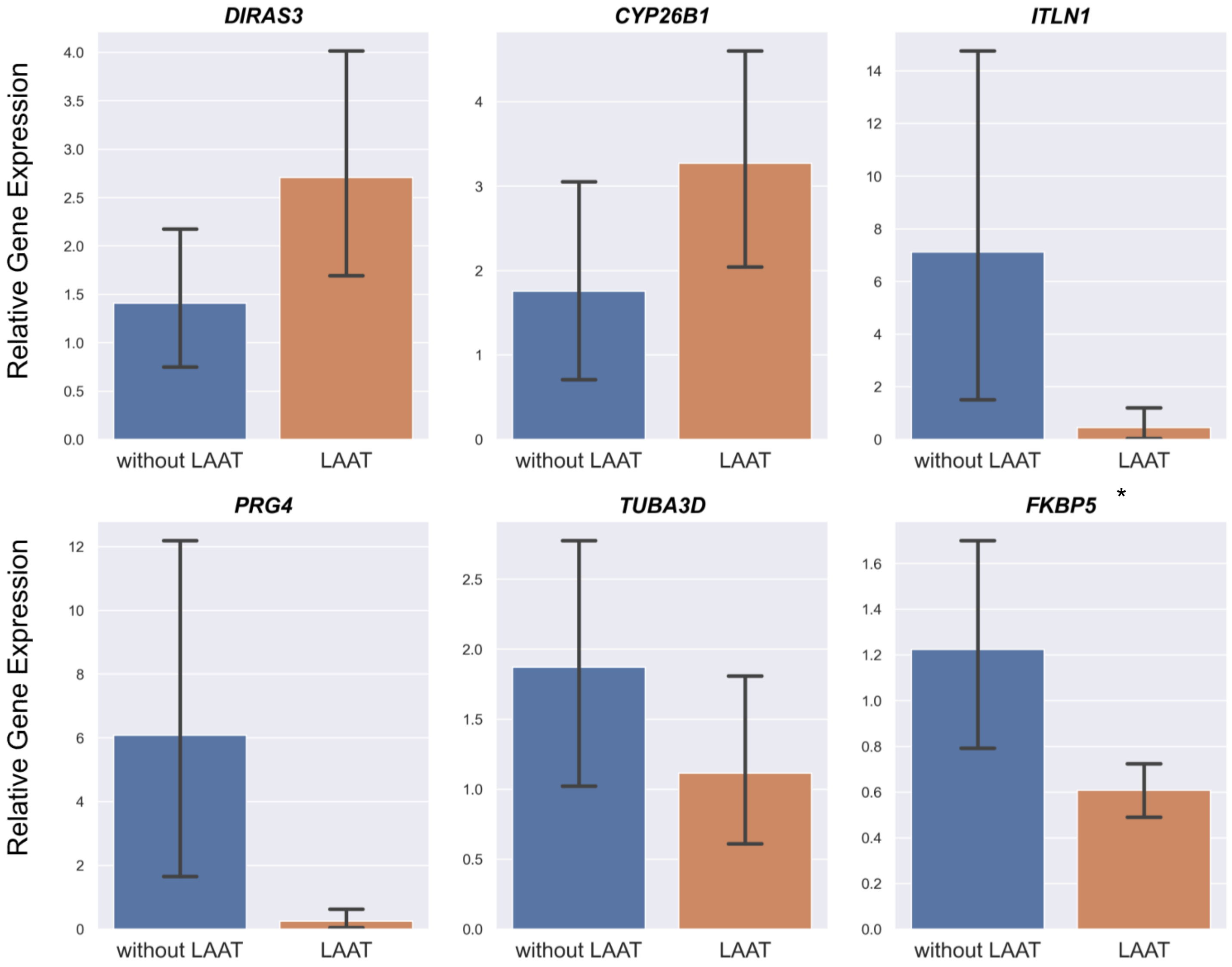

Supplementary Figure 1
Validation of RNA-seq Results by Quantitative PCR and in an Independent Sample Set

The mean relative gene expression ratios (LAAT/without LAAT) were as follows: 1.92 for *DIRAS3,* 1.86 for *CYP26B1*, 0.064 for *ITLN1*, 0.042 for *PRG4*, 0.60 for *TUBA3D*, and 0.035 for *FKBP5*.
*: $P < 0.05$
LAAT: left atrial appendage thrombus
