# Supplementary material for: RNA sequence analysis of differentially expressed genes in left atrial appendage thrombus
Source: J Thromb Thrombolysis. 2025 Oct 5;59(2):437–49. doi: 10.1007/s11239-025-03184-1 (PMC13018052; doi:10.1007/s11239-025-03184-1)
Supplement: Supplementary file 2 — Supplementary file2 (PDF 373 KB) [file 11239_2025_3184_MOESM2_ESM.pdf]

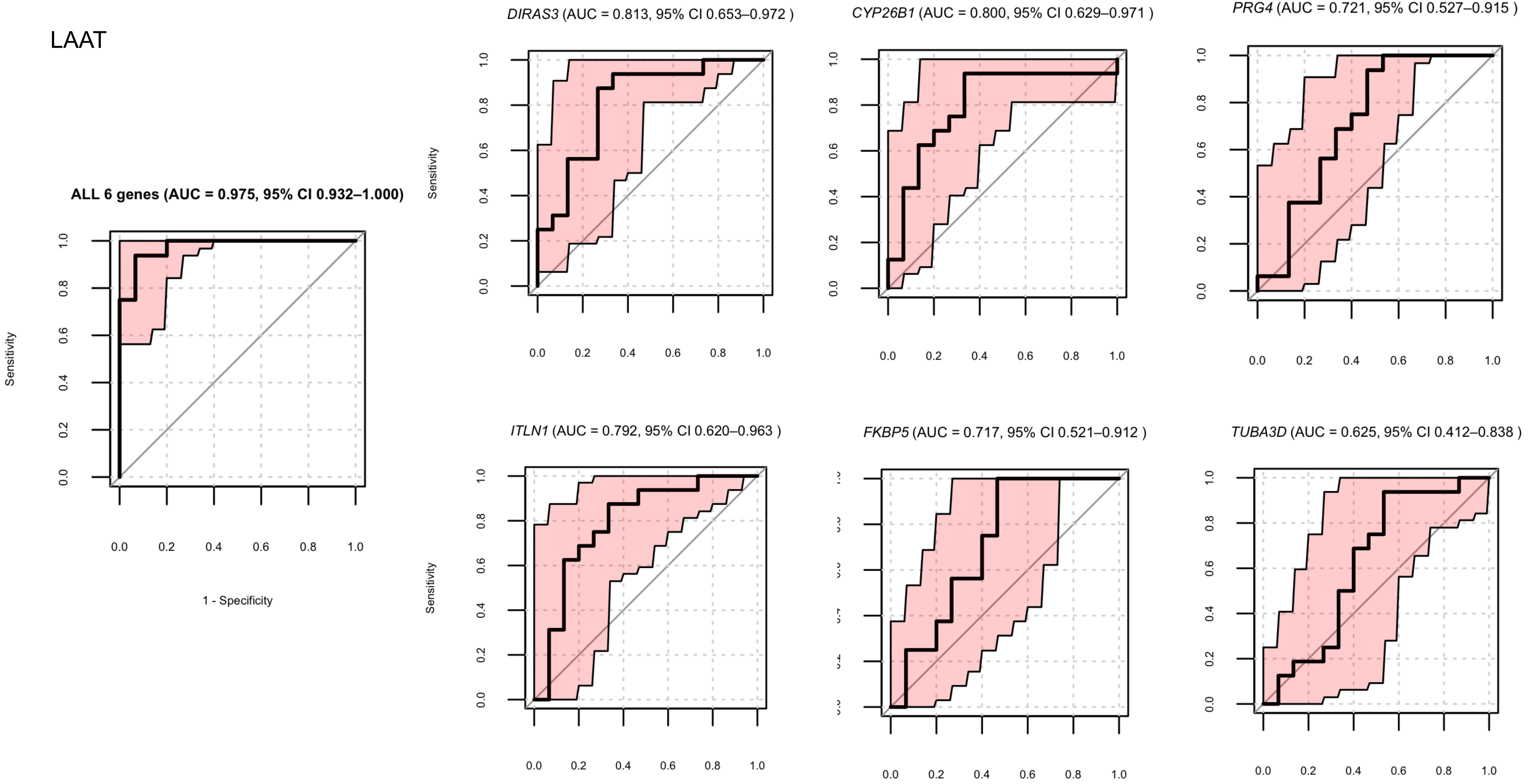

Supplementary Figure2:
ROC Curve–Based Assessment of Diagnostic Performance (LAAT)
The AUC for LAAT using all six candidate genes was 0.98 (95% CI, 0.93–1.00). Among these, the AUCs for the individual genes were as follows: *DIRAS3*, 0.81; *CYP26B1*, 0.80; *PRG4*, 0.72; *ITLN1*, 0.79; *FKBP5*, 0.72; and *TUBA3D, 0.63.*
