# Supplementary material for: RNA sequence analysis of differentially expressed genes in left atrial appendage thrombus
Source: J Thromb Thrombolysis. 2025 Oct 5;59(2):437–49. doi: 10.1007/s11239-025-03184-1 (PMC13018052; doi:10.1007/s11239-025-03184-1)
Supplement: Supplementary file 3 — Supplementary file3 (PDF 365 KB) [file 11239_2025_3184_MOESM3_ESM.pdf]

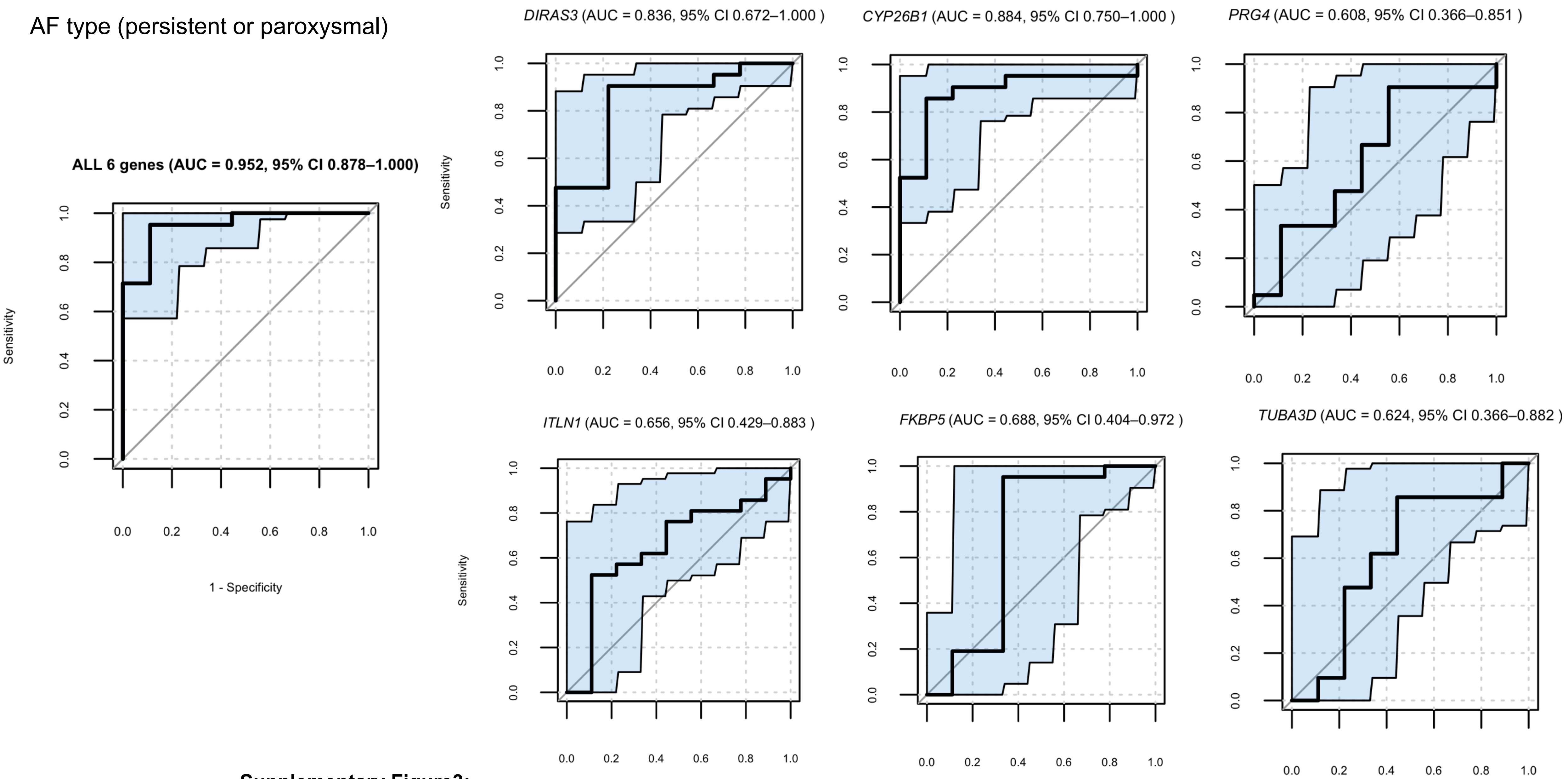

Supplementary Figure3:

ROC Curve–Based Assessment of Diagnostic Performance (AF type)

The AUC for AF type (persistent or paroxysmal) using all six candidate genes was 0.95 (95% CI, 0.88–1.00). Among these, the AUCs for the individual genes were as follows: *DIRAS3*, 0.84; CYP26B1, 0.88; *PRG4*, 0.61; *ITLN1*, 0.66; *FKBP5*, 0.69; and *TUBA3D*, 0.62.
