# Supplementary material for: RNA sequence analysis of differentially expressed genes in left atrial appendage thrombus
Source: J Thromb Thrombolysis. 2025 Oct 5;59(2):437–49. doi: 10.1007/s11239-025-03184-1 (PMC13018052; doi:10.1007/s11239-025-03184-1)
Supplement: Supplementary file 4 — Supplementary file4 (PDF 309 KB) [file 11239_2025_3184_MOESM4_ESM.pdf]

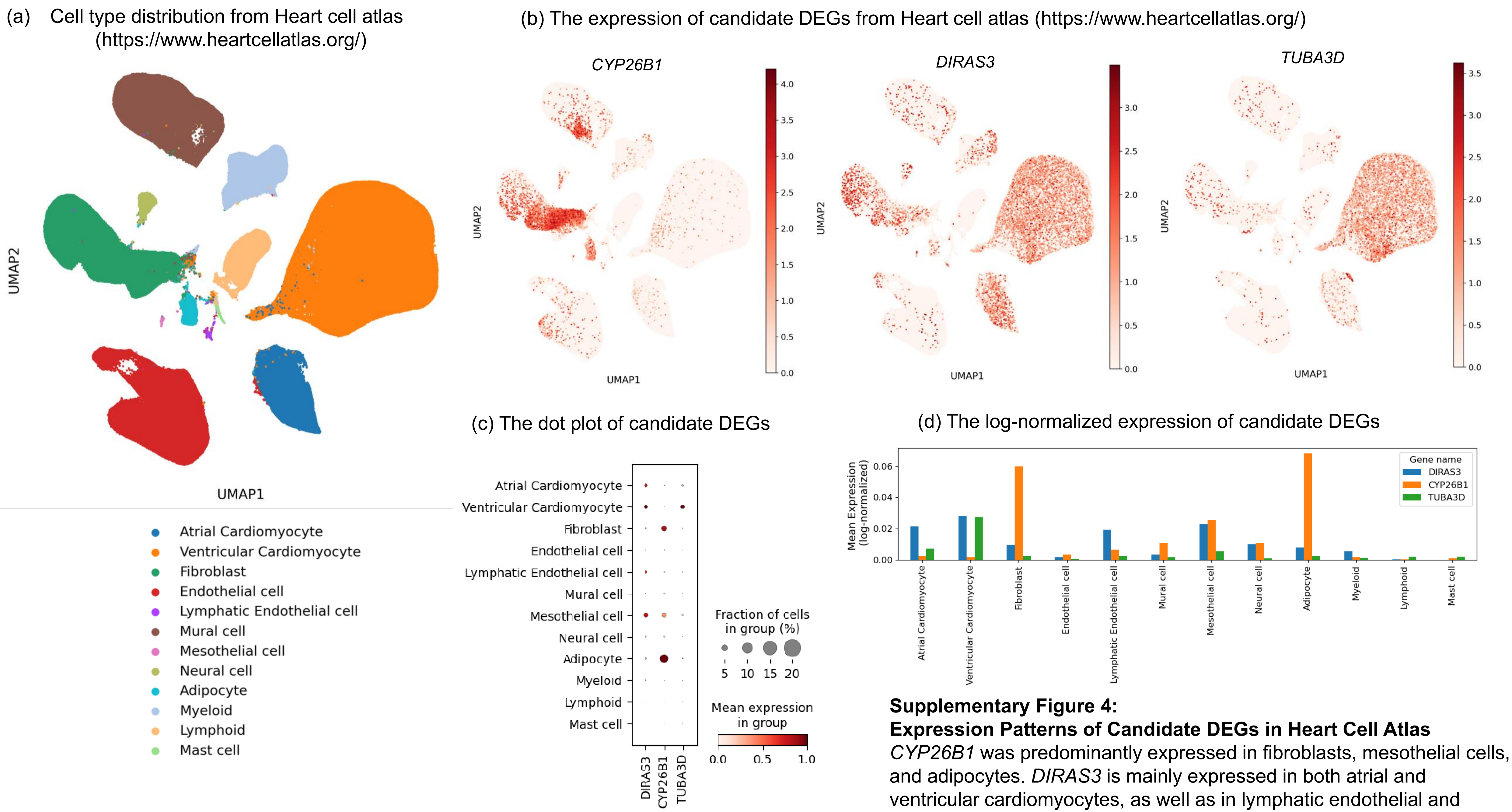

Supplementary Figure 4:
Expression Patterns of Candidate DEGs in Heart Cell Atlas
CYP26B1 was predominantly expressed in fibroblasts, mesothelial cells, and adipocytes. *DIRAS3* is mainly expressed in both atrial and ventricular cardiomyocytes, as well as in lymphatic endothelial and mesothelial cells. *TUBA3D* expression was expressed in ventricular cardiomyocytes
